# Evaluation of ChatGPT’s Real-Life Implementation in Undergraduate Dental Education: Mixed Methods Study

**DOI:** 10.2196/51344

**Published:** 2024-01-31

**Authors:** Argyro Kavadella, Marco Antonio Dias da Silva, Eleftherios G Kaklamanos, Vasileios Stamatopoulos, Kostis Giannakopoulos

**Affiliations:** 1 School of Dentistry European University Cyprus Nicosia Cyprus; 2 Research Group of Teleducation and Teledentistry Federal University of Campina Grande Campina Grande Brazil; 3 School of Dentistry Aristotle University of Thessaloniki Thessaloniki Greece; 4 Mohammed Bin Rashid University of Medicine and Health Sciences Dubai United Arab Emirates; 5 Information Management Systems Institute ATHENA Research and Innovation Center Athens Greece

**Keywords:** ChatGPT, large language models, LLM, natural language processing, artificial Intelligence, dental education, higher education, learning assignments, dental students, AI pedagogy, dentistry, university

## Abstract

**Background:**

The recent artificial intelligence tool ChatGPT seems to offer a range of benefits in academic education while also raising concerns. Relevant literature encompasses issues of plagiarism and academic dishonesty, as well as pedagogy and educational affordances; yet, no real-life implementation of ChatGPT in the educational process has been reported to our knowledge so far.

**Objective:**

This mixed methods study aimed to evaluate the implementation of ChatGPT in the educational process, both quantitatively and qualitatively.

**Methods:**

In March 2023, a total of 77 second-year dental students of the European University Cyprus were divided into 2 groups and asked to compose a learning assignment on “Radiation Biology and Radiation Protection in the Dental Office,” working collaboratively in small subgroups, as part of the educational semester program of the Dentomaxillofacial Radiology module. Careful planning ensured a seamless integration of ChatGPT, addressing potential challenges. One group searched the internet for scientific resources to perform the task and the other group used ChatGPT for this purpose. Both groups developed a PowerPoint (Microsoft Corp) presentation based on their research and presented it in class. The ChatGPT group students additionally registered all interactions with the language model during the prompting process and evaluated the final outcome; they also answered an open-ended evaluation questionnaire, including questions on their learning experience. Finally, all students undertook a knowledge examination on the topic, and the grades between the 2 groups were compared statistically, whereas the free-text comments of the questionnaires were thematically analyzed.

**Results:**

Out of the 77 students, 39 were assigned to the ChatGPT group and 38 to the literature research group. Seventy students undertook the multiple choice question knowledge examination, and examination grades ranged from 5 to 10 on the 0-10 grading scale. The Mann-Whitney *U* test showed that students of the ChatGPT group performed significantly better (*P*=.045) than students of the literature research group. The evaluation questionnaires revealed the benefits (human-like interface, immediate response, and wide knowledge base), the limitations (need for rephrasing the prompts to get a relevant answer, general content, false citations, and incapability to provide images or videos), and the prospects (in education, clinical practice, continuing education, and research) of ChatGPT.

**Conclusions:**

Students using ChatGPT for their learning assignments performed significantly better in the knowledge examination than their fellow students who used the literature research methodology. Students adapted quickly to the technological environment of the language model, recognized its opportunities and limitations, and used it creatively and efficiently. Implications for practice: the study underscores the adaptability of students to technological innovations including ChatGPT and its potential to enhance educational outcomes. Educators should consider integrating ChatGPT into curriculum design; awareness programs are warranted to educate both students and educators about the limitations of ChatGPT, encouraging critical engagement and responsible use.

## Introduction

### Background

The emergence of ChatGPT (OpenAI) in November 2022 represents the third significant technological breakthrough in information technology impacting education, following the introduction of Web 2.0 over a decade ago [1] and e-learning’s surge during the COVID-19 pandemic [2]. ChatGPT is an artificial intelligence (AI) tool that offers benefits and opportunities in higher education including increased student engagement, collaboration, personalized feedback, and accessibility. However, it is characterized by a limited database, posing challenges such as the restricted ability to answer medical questions and the potential for inaccurate and biased responses. There are also concerns regarding legal and ethical implications, plagiarism, and academic integrity [3-5].

The research on AI and its implementation in academic education is a prominent subject; a Google Scholar search for “artificial intelligence and dental education,” yielded 100,000 results and approximately 18,000 results for “ChatGPT and higher education” (on June 9, 2023). AI technology has evolved to unprecedented levels, transforming professions, revolutionizing workflows, and reshaping human-machine interactions. ChatGPT, the most recent milestone in natural language processing AI models, has been enabling advanced conversational capabilities and expanding the boundaries of AI-powered communication. Interest in ChatGPT applications encompasses both clinical practice [6,7] and higher education [3,8-11], with promising results.

### Relevant Prior Research

Within the higher education landscape, it has been suggested that dental curricula at universities need to be updated due to the AI paradigm shift [9,12,13]. This involves defining a fundamental dental curriculum for both undergraduate and postgraduate levels and establishing learning outcomes related to dental AI [8]. Cotton et al [3] and Halaweh [14] proposed strategies to ensure the ethical and responsible use of AI tools in higher education. Fergus et al [10] evaluated academic answers generated using ChatGPT, and Bearman et al [15] in their review on AI in higher education discussed the shifting dynamics of authority and the relationships among teachers, students, institutions, and technologies. Gimpel et al [16] in their extensive discussion paper proposed guidelines and recommendations for students and lecturers and urged the universities for a multistakeholder dialogue to implement efficient and responsible use of generative AI models in higher education.

Roganovic et al [17] performed a cross-sectional web-based survey among experienced dentists and final-year undergraduate students from the School of Dental Medicine, University of Belgrade, Serbia, to investigate their current perspectives and readiness to accept AI into practice. Responders, especially final-year students, showed a lack of knowledge regarding AI use in medicine and dentistry (only 7.9% of them were familiar with AI use) and were skeptical (only 34% of them believed that AI should be used in dental practice); the underlying reasons were fear of being replaced by AI, as well as a lack of regulatory policies, since students and—at a lesser degree—dentists were concerned that using AI could legally complicate the clinical practice [17].

Chan and Hu [11] reported different results in exploring students’ perceptions of generative AI and ChatGPT in teaching and learning through a web-based questionnaire; the study revealed a generally positive attitude toward generative AI, with students demonstrating a good understanding of this technology, its benefits, and limitations, despite its novel public appearance. Generative AI is a special category of AI designed to learn from the characteristics of its input and generate outputs with similar characteristics. In contrast to most AI models that perform specific tasks based on predefined rules and patterns, generative AI models use advanced algorithms to find the underlying patterns of the input data (eg, text, images, sounds, and videos) and “generate” entirely new content of the same type [11]. Students recognized the potential for personalized feedback and learning support, brainstorming, writing assistance, and research capabilities and stated they would integrate technologies like ChatGPT in their studies and future careers, but they were also concerned about becoming overreliant on them. They moreover expressed concerns about data accuracy, privacy, ethical issues, and the impact on personal development [11]. Students’ perceptions of the learning environment and the teaching strategies have a significant impact on their approach to learning and the learning outcomes (positive perceptions lead to a deep approach to learning), thus being of pedagogical interest to educators and institutions [11,18]. The influence of AI tools on students’ engagement and perceptions was investigated by Nazari et al [19]: they conducted a randomized controlled trial to examine the efficacy of an AI-powered writing tool (Grammarly) for postgraduate students and concluded that students in the intervention group demonstrated significant improvement in engagement (behavioral, emotional, and cognitive), self-efficacy, and academic emotions (positive and negative), domains that address learning behavior, which lead to self-development and underpin authentic pedagogy.

### Aims of the Study

Despite numerous publications about AI and large language models (LLMs), the majority involve discussion papers, viewpoint articles, and positions [3,13,16,20,21], with few being exploratory, cross-sectional, or questionnaire-based studies [11,17,19]. To our knowledge, so far, no experimental studies have been identified, wherein ChatGPT was in vivo implemented by students within the teaching process, and the outcomes were comprehensively evaluated.

Therefore, this study aimed to address this gap by implementing ChatGPT within the learning process and conducting a quantitative (differences between examination grades) and qualitative (thematic analysis of the free-text comments of the evaluation questionnaire) evaluation of the outcomes (mixed methods research study).

## Methods

### Ethical Considerations

The study’s research protocol was reviewed and approved by the Vice-Rector for Research and External Affairs and the President of the Institutional Committee on Bioethics and Ethics of the European University Cyprus.

### Study Design: Challenges

The study was conceptualized, organized, and refined in February 2023 and realized in March 2023. Of note is that ChatGPT appeared publicly on November 30, 2022; in March 2023, ChatGPT-3.5 was freely available (and was mostly used by the students), whereas ChatGPT-4 had just emerged (few students used this). The study was not a stand-alone research endeavor; instead, it constituted part of students’ educational activities embedded within the semester’s educational program. As this was the first attempt to implement ChatGPT in the educational process and there were no existing research studies in the literature to refer to, and adding to the limited knowledge on ChatGPT’s properties and limitations at the time, the authors encountered various challenges while organizing the research design. Therefore, to anticipate potential issues that could affect student learning or compromise the study’s outcomes, they conducted a systematic, forward-looking analysis of the research process, considering each step and taking proactive measures to mitigate any challenges or obstacles that may have arisen.

### Study Design: Implementation

The second-year dental students (77 students) of the School of Dentistry, European University Cyprus were randomly divided into 2 large groups and were asked to compose an assignment on “Radiation Biology and Radiation Protection in the Dental Office.” The subject of Dentomaxillofacial Radiology is taught through theoretical lectures, laboratory training, and practical training during 2 semesters, and students’ learning assignments are embedded within the lectures’ program as an alternative to traditional lecturing. Student learning assignments to replace lectures followed by in-class presentation and discussion is a methodology used within the “Dentomaxillofacial Radiology” module whenever the topic is suitable for such an approach. Students usually work collaboratively to perform the assignments by searching the internet for scientific reliable sources and compiling the results into a PowerPoint slide presentation, including the references they used. Students of both groups were asked to work in small subgroups to compose the assignments, where each subgroup would comprise 3-7 students, decided among them. It is worth mentioning that the European University Cyprus School of Dentistry is an English-speaking School, educating students from over 30 countries encompassing different ethnic, educational, and cultural backgrounds; therefore, the study’s sample could be considered diverse.

One large group would compose the assignment through literature research (the traditional method for assignments) and the other group would use the ChatGPT tool for the assignment (pose prompts and register the answers), also submitting a slide presentation. Students were given 1 month to deliver the assignment, and they were informed that they would present their presentations in class on a designated day.

Moreover, students of the ChatGPT group were encouraged to experiment with it; ask different questions; ask for videos, images, and internet resources; and in general to be creative, imaginative, and playful while using this new tool. Once they had the final AI content, they were advised to critically evaluate it by comparing it with the relevant content of a reliable scientific resource, such as a textbook or published article, and perform the necessary modifications to the AI output. After finishing the assignment, they were asked to complete an open-ended questionnaire individually (Multimedia Appendix 1), including questions about the usability, problems, opinions, proposals, and so forth, which was emailed to them, and which they would submit to the educator together with the assignment (ie, the PowerPoint presentation).

The AI Evaluation Questionnaire included 12 questions requiring free-text responses and was developed by the authors by combining questions from 2 sources: essays evaluation questionnaires retrieved in the scientific literature [22-24] and the questionnaire ChatGPT produced on the prompt “Can you develop 10 questions for a user to evaluate your performance on writing an essay?” Questions were combined and modified, they were piloted within a small student group other than the research groups, and they were finally amended as necessary. The free-text comments of the AI Evaluation Questionnaire were grouped into main themes and discussed (subjective and qualitative evaluation).

After students completed and submitted their projects via email, and on the designated day they would present the PowerPoint presentations in class, at the beginning of the session, they all had an unannounced blind knowledge examination (answered individually and anonymously, where they only indicated the group they belonged in, so that the educator could not relate the students with the answer sheets). The examination was developed by the authors and consisted of 10 multiple-choice questions (MCQs), which addressed the learning objectives of the topic. They were informed that the knowledge test was intended for the educator to identify whether the assignment had equipped them with the intended knowledge and whether there were any knowledge gaps to address. The results of the examination (examination grades) were compared among the 2 groups, that is, the literature research group and the ChatGPT group. Statistically significant differences between the groups’ grades were explored using the Mann-Whitney nonparametric test. Data analysis was conducted using SPSS (version 25.0; SPSS Inc), and statistical significance was set at *P*=.05 (objective and quantitative evaluation).

The final study design is summarized as follows:

- Students were randomly divided into 2 large groups (the ChatGPT and the literature research groups) and further into smaller groups.
- Literature research group performed the assignment by searching the internet and delivered it in PowerPoint format, including the references used.
- ChatGPT group (1) asked the LLM relevant queries and developed a PowerPoint presentation; (2) registered and reported on their interactions with ChatGPT, including the prompts and their modifications, the final outcome and its evaluation after comparing it with a reference text or book chapter; and (3) answered the AI Evaluation Questionnaire on their experience with the LLM.
- All students presented their learning assignments in class. At the beginning of this session, they undertook an unannounced knowledge examination of 10 questions.
- Data derived from the knowledge examination grades, the PowerPoint presentations, and the free-text comments of the AI Evaluation Questionnaire.

## Results

### Quantitative Results

**Figure 1:**
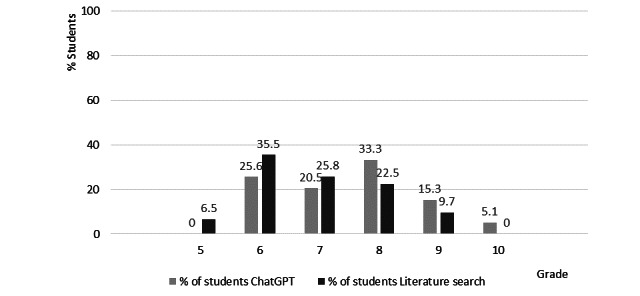
Students’ examination grades (% of students within each group).

Out of the 77 students, 39 were assigned to the ChatGPT group forming 9 subgroups and 38 to the literature research group forming 8 subgroups. Seventy students undertook the MCQ examination (7 students were absent) and examination grades ranged from 5 to 10 on the 0-10 grading scale. Figure 1 presents the number of students (percentages within each group) with their examination grades. We noticed that in the higher range of examination grades, that is, 8-10, the ChatGPT students outperformed the literature research students, while the opposite happened within the lower range of examination grades, that is, 5-7.

To check for differences between the ChatGPT student group and the literature research group, we performed the Mann-Whitney *U* test, which showed that students of the ChatGPT group (n=39; mean 7.54, SD 1.18) performed significantly better (*P*=.045) than students in the literature research group (n=31; mean 6.94, SD 1.12).

To foster inclusiveness and avoid discrimination, we deliberately chose not to perform statistical analyses regarding gender differences, as we also believe that gender diversity is not associated with the educational process or the educational outcomes. Education is offered equally to all students and any gender differences possibly found would not differentiate educational approaches for one gender or the other. Instead, we perceive this student cohort as representatives of their generation (Generation Z), a characteristic that is directly related to this study’s outcomes and could explain several findings. This concept is in line with the US National Institute of Health recommendations for gender-neutral language [25].

### Qualitative Results

#### Overview

Out of the 39 students of the ChatGPT group, 31 (80%) students answered the 12 questions of the AI Evaluation Questionnaire. The free-text answers to the questions were grouped into themes and discussed. Three main themes emerged.

#### Collaboration With ChatGPT and Problems Encountered

Although the majority of students were aware that ChatGPT had surfaced a couple of months ago in the digital world and some of them had already used it, this was the first opportunity they had to actually work with it and “officially” use it within their studies, and they enjoyed and appreciated this opportunity. They characterized it as a “powerful and versatile tool,” “intuitive and intelligent,” “revolutionary,” and “enjoyable to work with” and they thought this experience was “interesting and different from the regular assignments.” They stated that learning to use these AI tools would improve their future practice but emphasized that “you have to learn how to properly use it.” They appreciated its human-like answers, as these “do not make the user feel distanced from technology.” A student stated:

In the beginning I was afraid it was going to be too difficult to work with but as I was discussing with it I understood its greatness. I think it really is the future as it can help both education and research. I really did enjoy its human-like answers like when something was wrong it persisted like a human being for its accuracy as well as when it did not answer the question as it should like a lazy student.

Another student commented: “I enjoyed working with ChatGPT, because I got to learn and understand something that is going to be a part of the future.” Humanization of the LLM is worth noting: “He always understood what we wanted.” Textbox 1 shows examples of students’ prompts.

**Examples of students’ prompts to ChatGPT (exact copies).**

- How does radiation affect human health?
- What’s the difference between deterministic & stochastic effects of radiation?
- Is radiation exposure carcinogenic?
- Which are the radiation doses from common dental radiographic exams?
- Which criteria are used to reduce unnecessary radiographic exposure in dentistry?
- Can a pregnant employee continue to work in the dental radiology department?
- What is the importance of radiation biology? With references used
- What are the effects of radiation on cells and tissues? With references used
- What are the effects of radiation on the oral cavity? Rewrite the previous answer in a more elaborate way
- Make a chart about effective dose from diagnostic x-ray examinations focusing on the oral cavity
- Radiation biology, include references
- Measurements of radiology safety, include references
- Radiology protection in dentistry, include references
- How can we minimize the radiation exposure on dental staff, including references
- Why are radiation safety precautions necessary for the dentist
- Tell me how radiation can affect the human body
- Write me an essay discussing radiology safety and protection procedures in dentistry
- Can you explain radiation biology for medicine and dentistry in 400 words, include references
- Radiation exposure in dental office word limit 200-250 words. Include references
- Radiation monitoring in the dental office in 230-270 words include references
- Write me an essay of 400 words about the biology of radiation and provide references
- Write me a 300 words essay about radiation safety and protection in dentistry
- What are the risks associated with exposure to radiation?
- What are the modifying factors of irradiation?
- How does radiation exposure time and dose differentiate between adults and children in dental x-ray taking?

Not unexpectedly, students identified all the problems and limitations of ChatGPT, which are later described in detail in the literature. They identified the need to rephrase or detail the prompts to have a satisfactory output (“we learned quickly how to ask the questions to get a good answer”) and realized that if the same question was asked slightly differently the output was different (“by asking it 6 different questions, we wanted to get a better idea of what it changes on the text every time we put a new word or phrase the question differently”). They confirmed that some information was outdated, important content was missing, part of the answer was occasionally incorrect, links to references were nonexistent, and the links to videos were not working, although the LLM provided detailed and seemingly reliable information on the links and references (thus unknowingly identifying the “hallucination” effect of ChatGPT).

A student stated: “Mostly it understood our questions but it was not giving us that detailed and satisfactory answers as we anticipated according to our book.” Another student correctly noticed that “ChatGPT is not capable of having thoughts or opinions on its own, so it does not answer some questions that demand a critical-thinking answer.” Technical issues were also mentioned by some students, for example, “some days it was not opening and our conversation couldn’t be saved on the cloud” and “it ‘crushed’ sometimes mid-working.”

#### Quality of the Generated Outputs

Students found that the quality and depth of the information provided by ChatGPT depended on the quality and wording of the questions asked. As a student noticed:

I would not say that it demonstrated a very deep understanding of the topic, but I think with even more questions being asked, then the text could essentially show a deep understanding of the topic.

**Figure 2:**
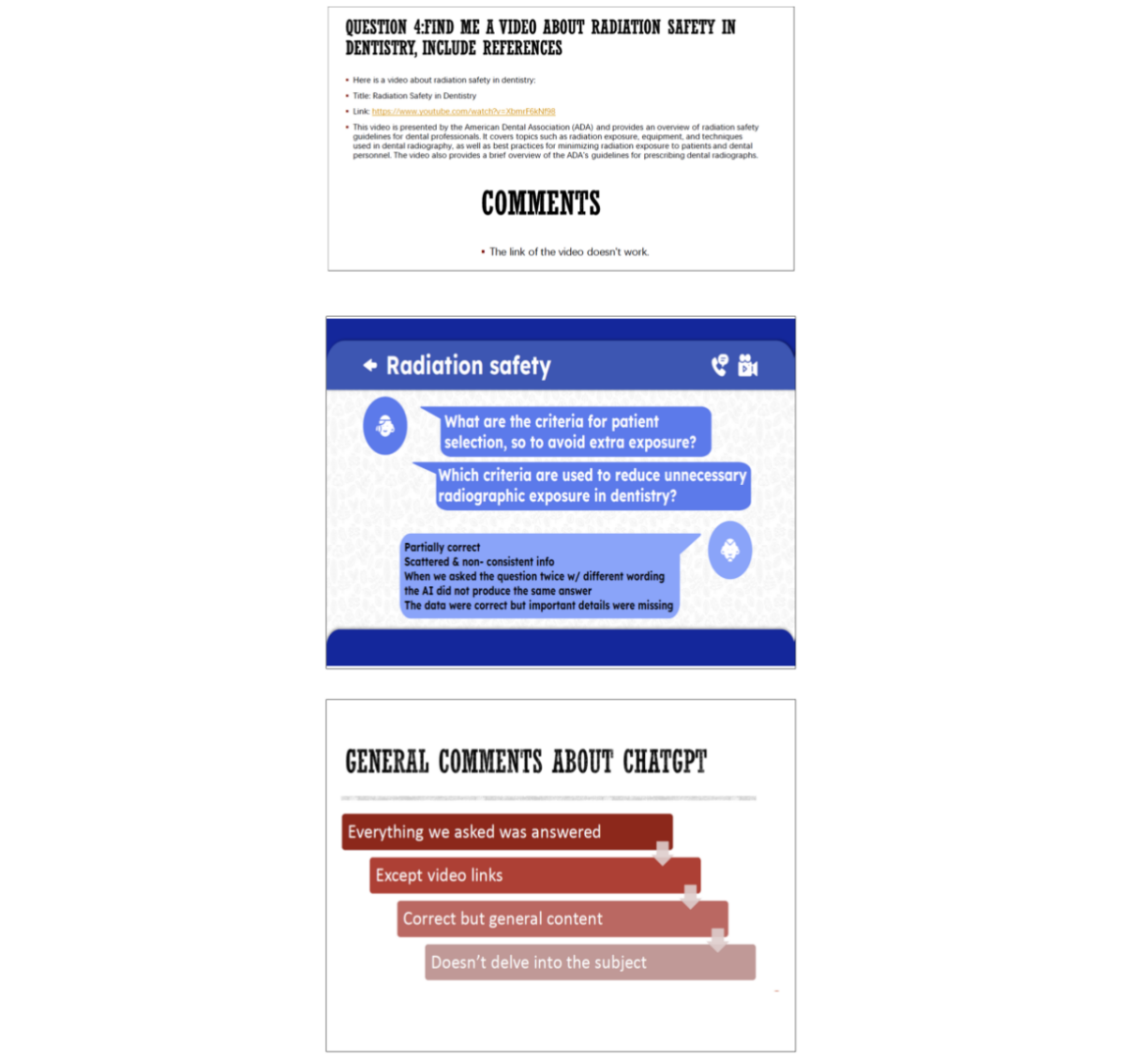
Examples of students’ slides depicting their interactions with ChatGPT.

Students quickly realized that with follow-up questions and rewording, they could guide the LLM to produce more detailed and in-depth answers: “it needed some guidance with follow up questions to further specify what we were asking for.” While comparing the output with a reference text, students reported that the answers were not detailed; sometimes included false data; and were brief, general, or superficial; nevertheless, the key points were evident. A student concluded that “ChatGPT is more than enough in order to understand and have a general idea about the main points of the matter being discussed” and another student thought that “I will find more details by going and searching online or in books.” They expect ChatGPT to improve in the future and be able to provide videos and images because “they are helpful in understanding a topic and provide a more effective way to retain information as well” and also to be able to browse external resources outside its stable database (Figure 2).

They evaluated the language as appropriate for a scientific document, understandable, and explanatory, and they indicated that when references were asked for, the language was even more formal and academic: “It is fascinating how the AI provides understandable answers in a scientific manner.” However, they encountered problems with the references, as in some occasions, ChatGPT denied to supply them, while in other instances, the references were incorrect. A student described:

The AI was continuously denying to give us relative references but after reforming our questions we eventually got our answer. The references it used were accurate scientific resources found on its stable database like the American Dental Association.

Another student stated that “We used chat GPT 4 so all our references were sufficient and up to date” (apparently overestimating ChatGPT-4’s currentness, as it has the same cutoff date as ChatGPT-3.5). The majority of students evaluated the references as relevant, sufficient, reliable, and up-to-date; however, they also recognized the limitations of the LLM, thinking that “it is under construction so not all its answers are up to date and sufficient information is only provided up to a certain point in time.”

#### Exploring Additional Possibilities and Predicting the Future

**Figure 3:**
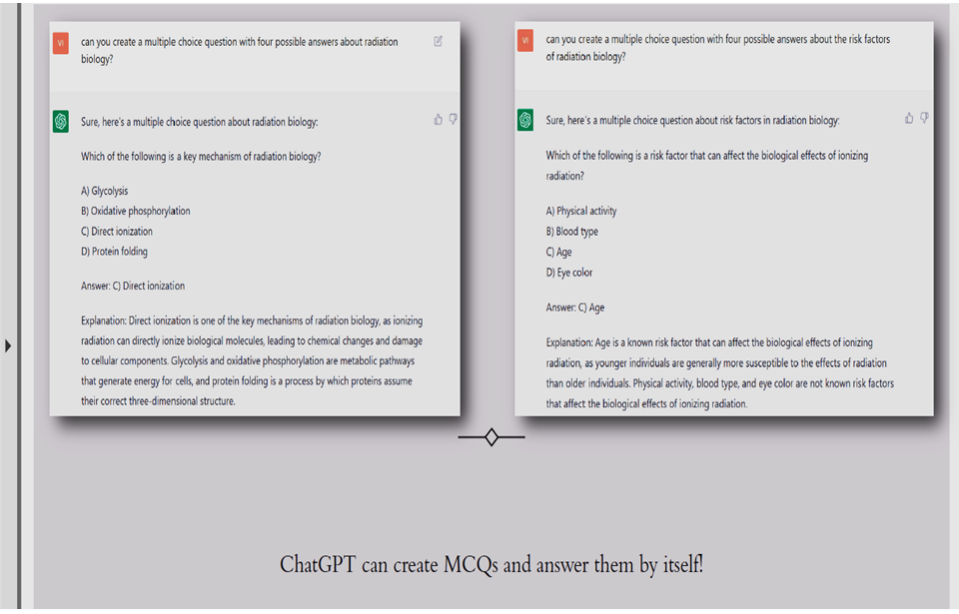
Multiple-choice questions created by ChatGPT. MCQ: multiple-choice question.

Students experimented with ChatGPT, asking it to provide images and videos, and create MCQs, charts, bullet point summaries, and presentation templates, for example, “we asked about multiple choice questions and the answers were actually impressive” (Figure 3). Students were imaginative and resourceful, and they were disappointed when their request was not realized:

I asked from it to provide me some explanatory images related to our topic, but it was not able to do so. I think this is a crucial disadvantage, as images give depth and context to a description and provide a much more immersive experience than writing alone.

Two student groups—comprised of technologically very experienced students—surprised the authors when they skillfully bypassed the inability of ChatGPT to produce PowerPoint presentations by asking it to write a programming code:

We used the AI for the generation of a PowerPoint. Since it cannot on its own generate PowerPoint Slides we asked it to generate a VBA code for the PowerPoint. That code was copied and then pasted to the ‘Developer’ section of the PowerPoint. As a result we got a beautiful but not so detailed presentation of our topic.

This process enabled the instant transfer of ChatGPT’s output within a PowerPoint slide presentation created by ChatGPT. Among the future applications of ChatGPT, students included the use in dental education, for example, for the creation of MCQs, summarizing a topic, lecture revision, helping students better understand a theory or concept, assignments and projects, laboratory reports, questions about law and ethics, communication with patients, and more. A student proposed:

Virtual patient consultations: ChatGPT could be used to simulate patient consultations for dental students. Students could practice various scenarios, including patient history taking, explaining diagnoses, and treatment planning.

Continuing education could also avail from the opportunities ChatGPT and LLMs offer:

Education that never ends: ChatGPT may be utilized to give dental professionals continual education. For dental professionals to keep current in their field, faculty might create modules containing the material they need, and ChatGPT may offer engaging tasks and tests to reinforce the learning.

Considering dental practice, students proposed that ChatGPT could be used to educate and solve problems for the dentist, for example, when “the dentist has a mind block” or when the dentist “seeks information about new dental materials and techniques”; also for treatment plans, schedule creation, and oral hygiene info; and for patient education “through integrating the model into a dental practice’s website or patient portal.”

For research and scientific publications, students thought it “can be useful to use it synergistically with your own research,” but “you should always double-check the information” and “keep in mind the plagiarism, using the information provided appropriately.”

Finally, students admitted that ChatGPT has drawbacks such as a limited database, incapability to access external web resources and provide images and videos, inaccurate links, and the need to verify the information generated. They thought that “it should be used with caution” and that “AI still needs to evolve,” so that it will become “an incredibly smart, effective, and powerful tool that can help the scientific community.” They realized that “the power it holds is unpredictable and the work of doctors could be compromised” and feared that “maybe we will live one day that AI robots could even replace dentists.” A student eloquently summarized ChatGPT’s past, present, and future:

After many years of research and after many science fiction movies about the power of AI and its impact on society I have come to the conclusion that this kind of AI can only help and do no harm. AI like ChatGPT that is available to the public and gives sufficient and accurate responses can give us hundreds of possibilities, even at dentistry. But I really don’t know this exact ChatGPT with its limited dental references can influence the field of dentistry. I can though imagine a more resourceful AI where it uses PubMed or Research Gate to generate its responses that would really elevate the level of dental education and research. What if a curious dentist had the million dollar question answered in milliseconds by the AI?

## Discussion

### Overview

In March 2023, a total of 39 dental students who are 20 years of age, through composing an educational assignment, identified the capabilities and limitations of the recently introduced ChatGPT and explored various possibilities; used it to write MCQs and programming codes; proposed future applications in education, research, and dental practice; and outperformed their peers in the knowledge examination.

### Results Explained and Compared

The quantitative results, that is, the examination grades, demonstrated that all students performed well (their grades fell within the middle and high ranges of the grading scale) and no students underperformed (no grades in the low ranges of the scale), while ChatGPT group students outperformed their literature research group peers. Since the examination occurred with no prior notice to the students, it directly reflects the knowledge acquired and retained through the project’s creation. Students’ good performances on the examination could be related to the format of the project in connection with their generational traits: all students socially belong to the Generation Z cohort (born between 1995 and 2010), so they are the first true “digital natives” [26], having grown up with smartphones, social networks, apps, and streaming content as part of the daily routine [27]. They are considered tech-savvy, mobile-driven, collaborative, and pragmatic [28,29] and possess a natural facility with digital tools and an interest for everything digital. Motivated by the opportunity to use the internet and work collaboratively, students immersed themselves in the project and explored it in depth, and this applies even more to the ChatGPT group students who were excited and curious to test this new digital tool. The enhanced learning observed with the ChatGPT students can be also attributed to the increased “time on task” for these students, as they had to spend more time asking and reasking the questions, evaluating the answers, correcting, and complementing them in comparison to their peers who had clear and readily available results from the relevant scientific literature. Additionally, ChatGPT group students had to work more than their fellow students with the learning material at a higher cognitive level and constantly apply critical thinking while experimenting with various questions and answers, comparing, and synthesizing them—an element that also enhances deep learning and results in enhanced performance [30].

The AI Evaluation Questionnaire provided insight into students’ opinions, evaluations of ChatGPT, the problems encountered, and their future estimations. Students demonstrated their prescience by providing remarks in concordance with those found in later-published articles; the latter were accessed by the authors after the research was concluded and while composing this study. Students evaluated their learning experience with ChatGPT as interesting, enjoyable, and engaging [19] and appreciated its user-friendly interface and the possibility of arguing with it [4,16]. They assessed the generated content as overall correct and sufficient [7,31], although often providing a general overview of the subject [5], as well as not demonstrating a deep understanding of the context [32-34] nor thinking critically [10,35]. They first-hand identified the need for carefully created questions [36] and critical analysis of the answers [14,36], and they urged for cautious and responsible use [4,6]. In agreement with Chan and Hu [11], they are ready to embrace this new technology but in a collaboration where people maintain control and are not replaced by AI [17,20,37,38]. Finally, in line with the literature, they attributed “anthropomorphic” qualities to the language model (1 student referred to ChatGPT using the gender pronoun “he”), possibly explained by the establishment of a personal connection between the student and the language model while engaging in human-like conversations in combination with student’s own gender-related perceptions and interaction style [39].

Students proposed possible applications of ChatGPT in education for revisions, MCQ creation, personalized learning, writing essays [3,4,20,37,40], and continuing education [38], as well as in research and clinical practice [4,6,12]. Nevertheless, students thought that the LLM must evolve to provide images, videos, accurate and relevant citations, and browse the internet [31,41,42].

Numerous publications thereafter examined the LLM’s limitations that had been already identified by the students: incorrect answers and outdated content [10] possibly due to its limited data set [37,38,43], the possibility for fabricated information and hallucination [44], false citations and links leading to nonexistent sources [38,44,45], inability to browse the web [41], and risks for plagiarism [3,46].

This research materialized Kung et al’s [31] concluding remarks that “the utility of generative language AI for medical education must be studied in real-world learning scenarios with students, across the engagement and knowledge spectrum” since ChatGPT was embedded within the educational process, thus producing authentic and relevant results. The quantitative and qualitative outcomes of this study indicate that this cohort of Generation Z students is capable of adapting quickly to new technologies and ready to use LLMs such as ChatGPT in the learning process—while acknowledging their limitations—particularly when these tools are integrated within a pedagogical framework that fosters creativity and autonomous learning. Educators on the other hand seem to have limited technological knowledge, skills, and pedagogical expertise to assess AI applications and successfully integrate them into education [12,47]; therefore, they should pursue professional development to develop new skills related to AI understanding, possibilities, and implementation [15,40,48,49].

### Pedagogical Aspects

All second-year students were asked to explore the topic of “Radiation Biology and Radiation Protection in the Dental Office” and develop assignments to be presented in class as PowerPoint presentations. Questions and knowledge gaps were covered during the in-class presentations by the instructor and not infrequently by their peers. This approach is consistent with the “flipped classroom” concept, an educational methodology that research has shown to engage students in the learning process, promote autonomy and self-regulation, allow for higher-order thinking, improve student satisfaction, and increase academic performance [50,51]. Another element of pedagogical interest is the small group collaborative work to develop the assignments. Collaborative learning has the potential to promote deep learning, which is essential for understanding complex concepts particularly in science education, through students’ meaningful interactions and constructive debates [52]. Scager et al [52] reported that effective collaboration is achieved when students undertake a challenging, complex task, and they succeed in creating a new and original output. Such tasks applied in higher education build a sense of responsibility and shared ownership of the output and the collaborative process, and this sense was indeed apparent in the students of this study within and during their oral presentations.

An additional pedagogical element is the learning assignments as a method for self-learning and knowledge acquisition. Learning through assignments has been reported to be preferred by students: in the study of Warren-Forward and Kalthoff [53], 79% of the students reported that the assignment on magnetic resonance imaging safety was both a positive learning experience and provided an understanding of the topic. Writing assignments enhance retention of knowledge; when assignments include reflective thinking, for example, when students have to evaluate and synthesize information (as happens in this study), higher-order (critical) thinking is also enhanced as students work at a higher cognitive level [30].

The innovative pedagogical aspects of this study (flipped classroom, learning assignments, and group learning) constituted a supportive environment for students of both groups to demonstrate their skills, achieve the learning objectives, and produce valuable results. While this pedagogical approach may cater more to certain types of learners, it remains pertinent for younger generations, who prefer active and collaborative learning.

### Study Design: Tackling the Challenges

Of interest would be to communicate herein the challenges faced during designing the research process, as the ChatGPT environment was largely unknown at the time, and obstacles and drawbacks had to be identified and resolved ahead through a step-by-step prospective analysis of the sequence of events.

For example, a concern that had to be addressed ahead was the fact that the subject was unknown to the students and they would not know whether the output was scientifically correct or incorrect, comprehensive or incomplete because they would not have an exemplary scientific text to compare it with, as they would rely solely upon ChatGPT’s answers. To address this, they were advised to compare the outcome with the relevant content of a recommended textbook (or other reliable source of their choice), critically evaluate the quality of the AI outcome, and perform the necessary amendments to complement or correct the AI results. The comparison should be included either within their presentation or within the AI Evaluation Questionnaire. This process would additionally ensure the achievement of learning objectives. In line with this process and at a later time, Chung [48] proposed in his article published in April 2023 that “instructors should teach students to use other authoritative sources (eg, reference books) to verify, evaluate, and corroborate the factual correctness of information provided by ChatGPT.”

Another concern arose about elucidating students’ engagement with ChatGPT: since the output of ChatGPT would be texts in slide format (similar to the ones of the literature research group), the educator (one of the authors) could evaluate these texts or slides for accuracy and comprehensiveness but could not comprehend whether they were generated following single or multiple attempts, posing differentiated or follow-up queries; therefore, the time and effort spent on the research process and the learning path could not have been assessed nor would the capabilities and drawbacks of the LLM be revealed. To address this concern, the ChatGPT group students were asked to register and report all their interactions with the LLM (including the number of prompts, the modification of prompts, the queries about references, images, and the underlying reasoning); thus, the educator could evaluate the cognitive effort they put in the assignment and the critical thinking applied until a satisfactory result was achieved. Furthermore, this would provide valuable insights into comprehending the usability and operational characteristics of the LLM. Adding to this, the AI Evaluation Questionnaire was a useful means to draw information on student-LLM interactions.

In accordance with the above procedure determined by the authors and in affirming their decisions, Halaweh’s study [14] published in April 2023—2 months after the development of this study’s design and 1 month after its implementation—precisely described the same process when discussing the strategies for successful implementation of ChatGPT in education. It seems that future literature confirmed the authors’ study design overall.

### LLMs in Higher Education

Given the study’s results and in agreement with the relevant literature, the authors would suggest that higher education institutions and dental schools could consider updating their curricula, policies, and teaching methods to prepare students for an AI-driven future, by including education on and with AI tools and LLMs [8,45]. Within this context, faculty professional development seems urgent to increase their skill level and AI understanding, for example, through peer support, mentoring, and sharing good teaching practices [36], as most educators have limited knowledge and skills to assess and efficiently use AI applications [12]. The introduction of LLMs into education will offer opportunities to improve its efficiency and quality: improved student performance, personalized learning, targeted and immediate feedback, increased accessibility, creativity and innovations, student engagement, lesson preparation, collaborative activities, and evaluation [4,40,54-56]. From the pedagogical perspective, students using LLMs have the potential to develop new competencies including 21st-century soft skills, such as self-reflection abilities, problem-solving skills, creative and critical thinking, and collaboration, thus becoming motivated and autonomous learners [3,4,16,33,49]. Moreover, as AI technology evolves and gradually integrates within the educational process, the conventional pedagogical theories may not be relevant nor sufficient to support the teacher-student-technology relationship, as technology profoundly alters the way students learn and engage with the content and the teacher; innovative pedagogies will be needed, such as the “entangled pedagogy” Fawns [57] proposed to contextualize students’ learning in a world where AI is increasingly prevalent [15,16].

To respond to the AI paradigm shift, higher education institutions, educators, and students must engage in constructive dialogue to develop policies, guidelines, and training opportunities for the implementation of innovative technological tools in the teaching process [16,34,55]. Despite the current weaknesses that limit their implementation, LLMs will likely improve in the future in terms of performance, scalability, and quality of responses, as well as through fine-tuning for specific tasks, customized use cases, and search engine connection [4,16,31,58].

### Limitations and Strengths

The small number of students who participated in this study (77 in total and 39 in the ChatGPT group) in 1 dental school can limit the extrapolation of the results. Students’ digital literacy is also of relevance: students who participated in this research were mostly tech-savvy, whereas students in other schools or universities may be less familiar with digital technologies; thus, results would not apply to them [17]. In addition, some findings (particularly the qualitative ones) may be outdated at the time of publication, as LLMs constantly evolve and new LLMs have been introduced since the research was conceptualized and implemented. For example, Google Bard and Microsoft Bing claim to have live access to the internet, a capability highly appreciated by the students; ChatGPT has since evolved its algorithms, with results being more accurate and relevant. Some elements of the study design could have been further explored; for example, students’ assignments could have been graded and compared, but since assignments’ grading was not included in the semester program of the module, this was not performed. In any case, the importance of this study lies in the fact that this was a very early attempt to implement legitimately and in vivo a language model in the teaching process as a partner in learning, in contrast to the large number of publications perceiving ChatGPT as a partner in cheating and academic dishonesty [12,59,60]. Another strength would be that it revealed aspects of the language model-students’ interactions during the learning process, which indicate that this emerging relationship is yet to be explored, and updated pedagogical frameworks are needed for this purpose.

### Conclusions

ChatGPT was implemented in real-life undergraduate dental education and was evaluated. Students using ChatGPT for their learning assignments performed significantly better in the knowledge examination than their fellow students who used the literature research methodology. The AI questionnaire answered by students revealed the capabilities and weaknesses of the language model, as identified later in the scientific literature. Students enjoyed working with this tool and explored different options and possibilities, indicating that they are technologically knowledgeable and capable of adapting to new technologies, both in education and in future clinical practice. LLMs such as ChatGPT have the potential to play a role in education, underpinned by solid pedagogies.

## Appendix

Multimedia Appendix 1 AI evaluation questionnaire.

## Abbreviations

AI: artificial intelligence
LLM: large language model
MCQ: multiple-choice question
