# Supplementary material for: Evaluation of ChatGPT’s Real-Life Implementation in Undergraduate Dental Education: Mixed Methods Study
Source: JMIR Med Educ. 2024 Jan 31;10:e51344. doi: 10.2196/51344 (PMC10867750; doi:10.2196/51344)
Supplement: Multimedia Appendix 1 [file mededu_v10i1e51344_app1.docx]

## Appendix

**AI EVALUATION QUESTIONNAIRE**

1. GENERAL INFORMATION

Name:

Email:

Digital knowledge: How experienced and skillful are you in digital applications and overall the digital world (internet, search engines, digital manipulations, etc)? (Underline or bold your choice)

very experienced

experienced enough

moderately experienced

barely experienced

no experienced at all

1. CHATGPT EVALUATION

Please reply the following questions in as much detail as possible.

1. Did you enjoy working with ChatGPT? Was it an interesting experience? Please explain
2. Did you encounter problems? Please explain
3. Did it understand your question? How many attempts (i.e. questions asked) did you perform until you had a satisfactory text? Please write all the questions you asked.
4. Did you compare the ChatGPT essay with a “model” text? With which one?
5. What did this comparison show? Was the ChatGPT essay comparable to the “model” text and did it include all the main points? Please explain
6. Did the essay effectively address the given topic? Please explain
7. Did it demonstrate a deep understanding of the topic and related concepts? Please explain
8. Was the language professional and appropriate for a scientific document?
9. Were there any areas it could have provided more information? Which ones?
10. How well did it incorporate relevant research and literature into the essay? Were the references included relative, sufficient and up-to-date? Please explain
11. Did you search ChatGPT for other options than questions-answers (text writing)? What were the results? Please explain
12. How do you think this AI application could be used in dental education? Please write your ideas
